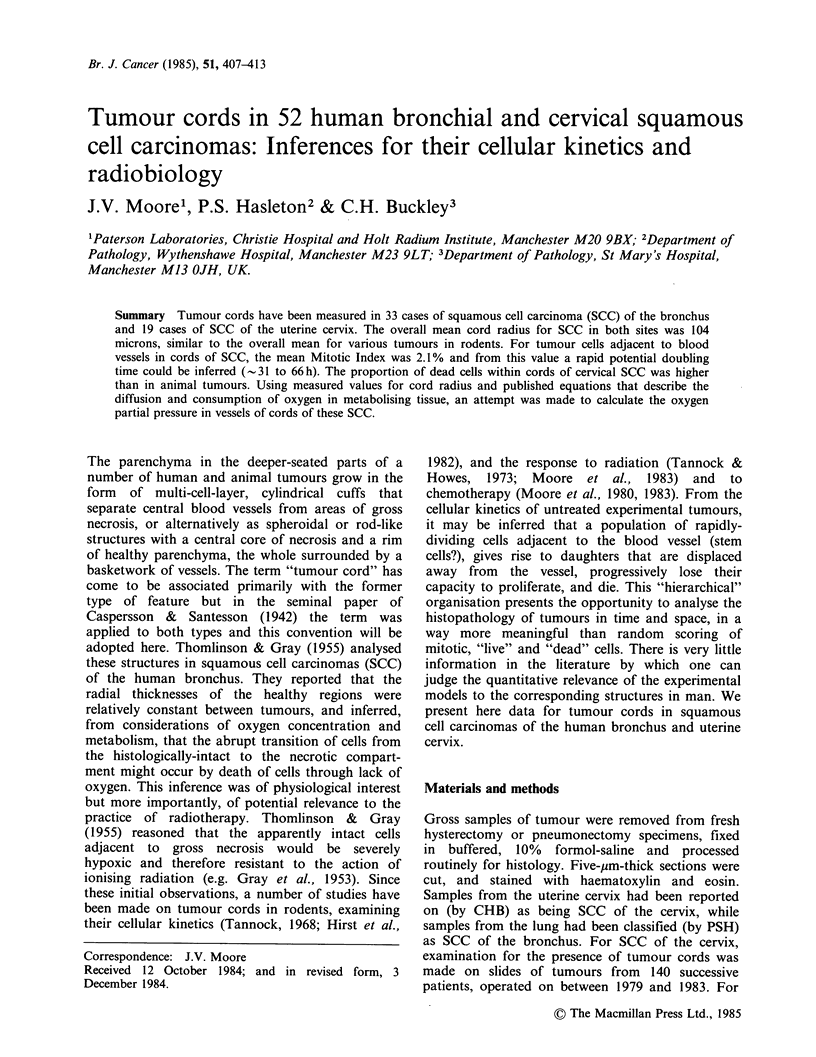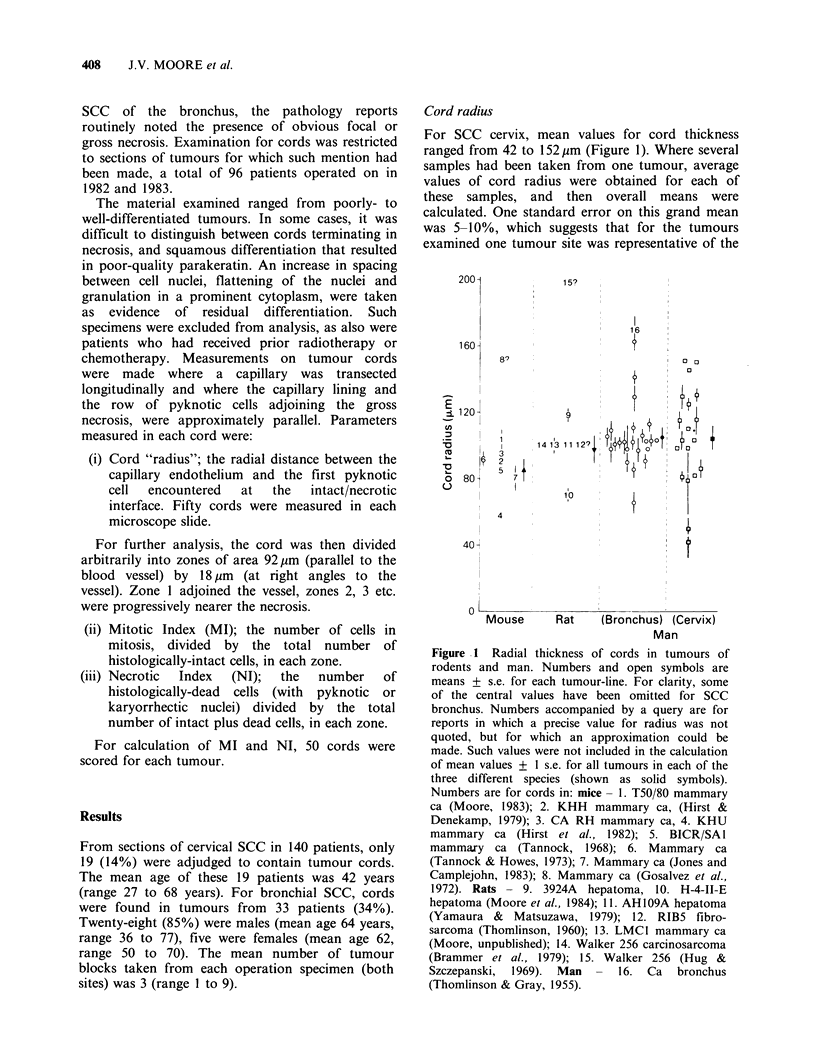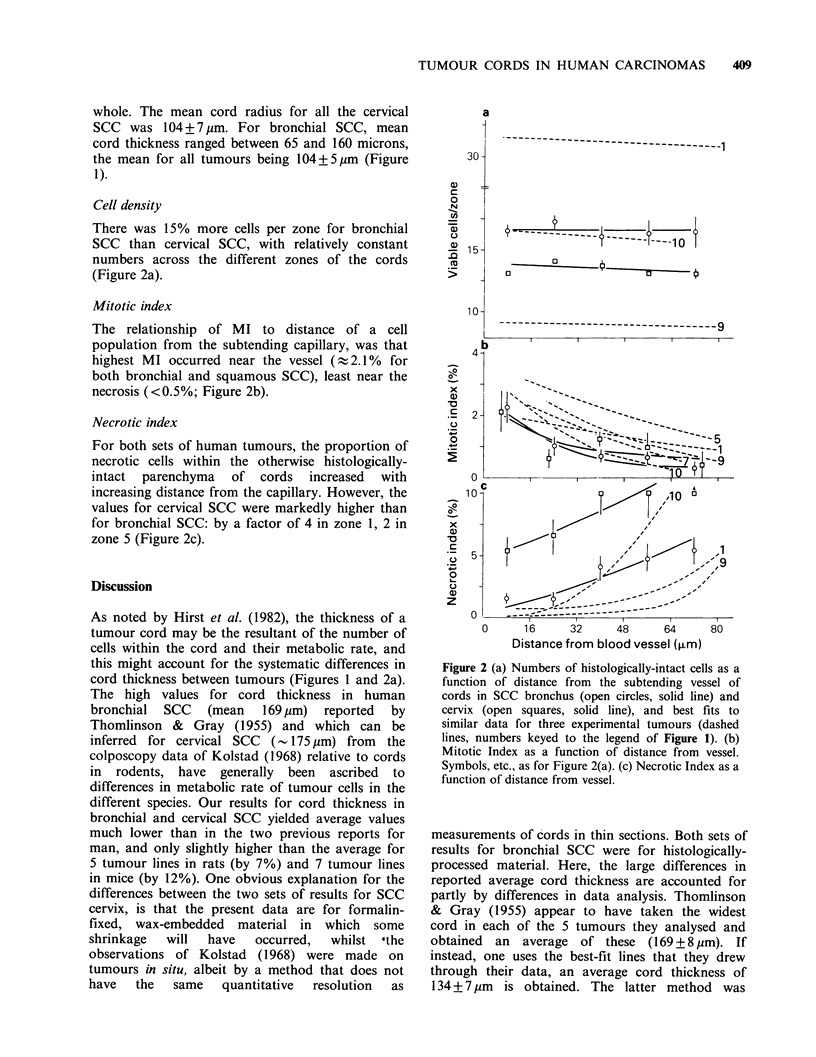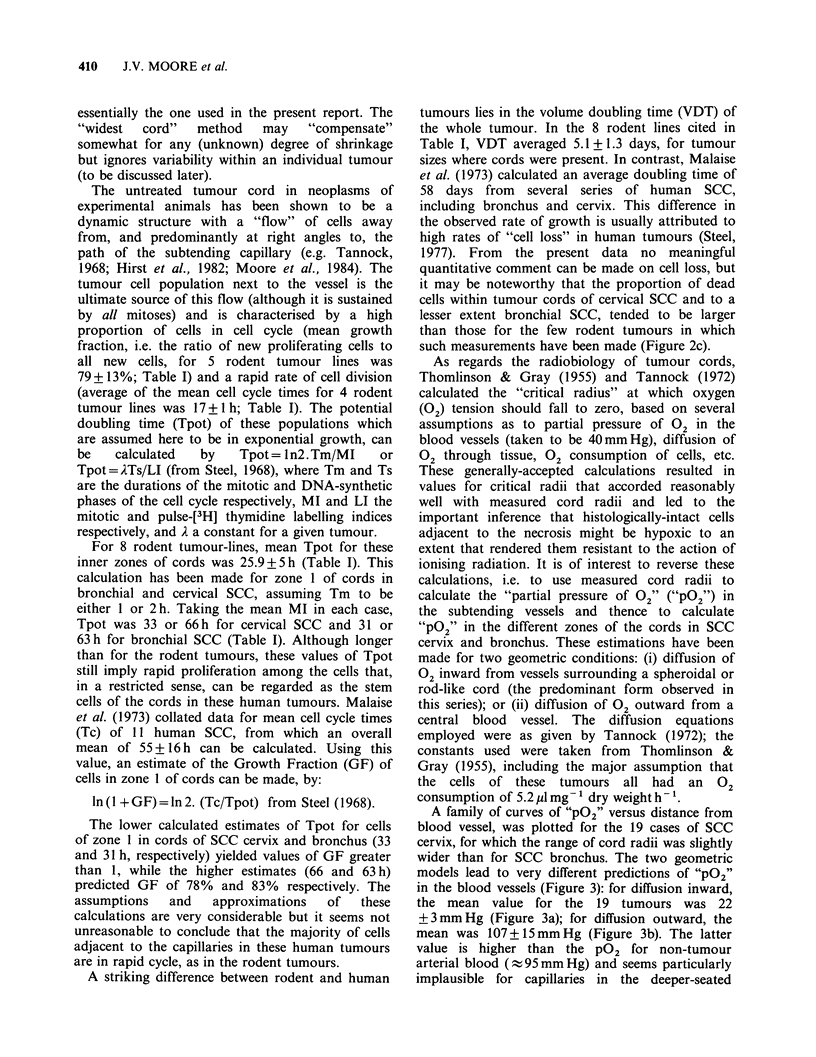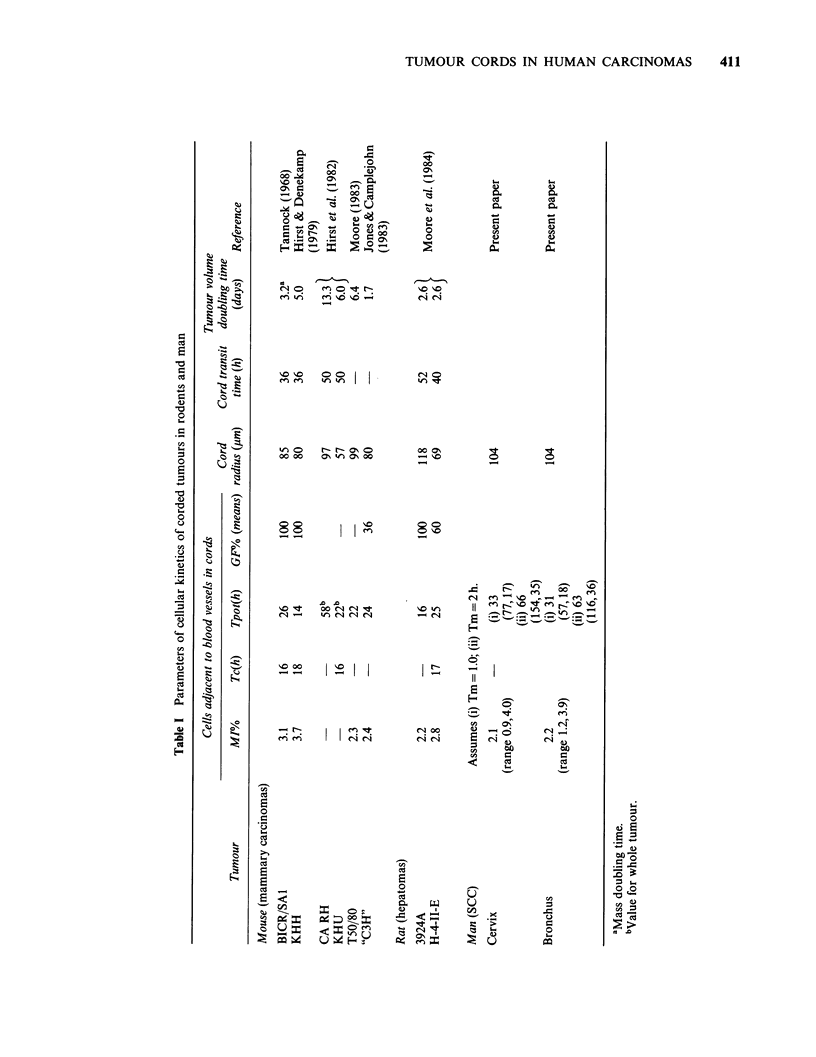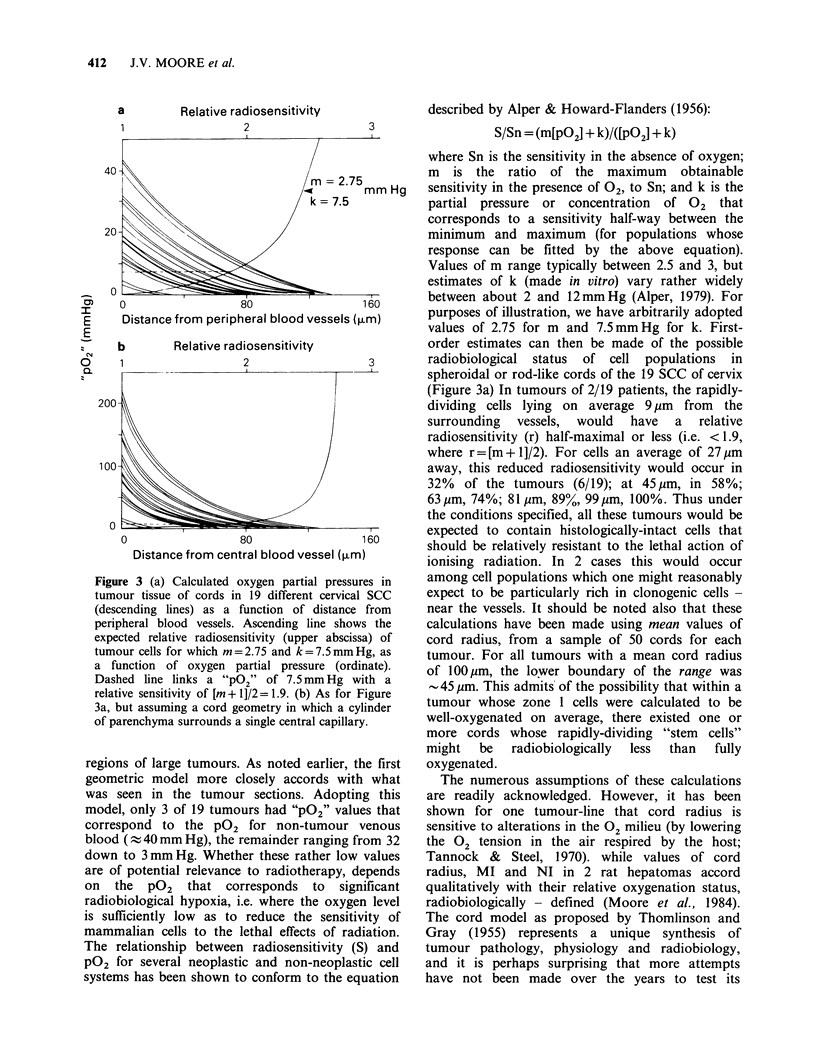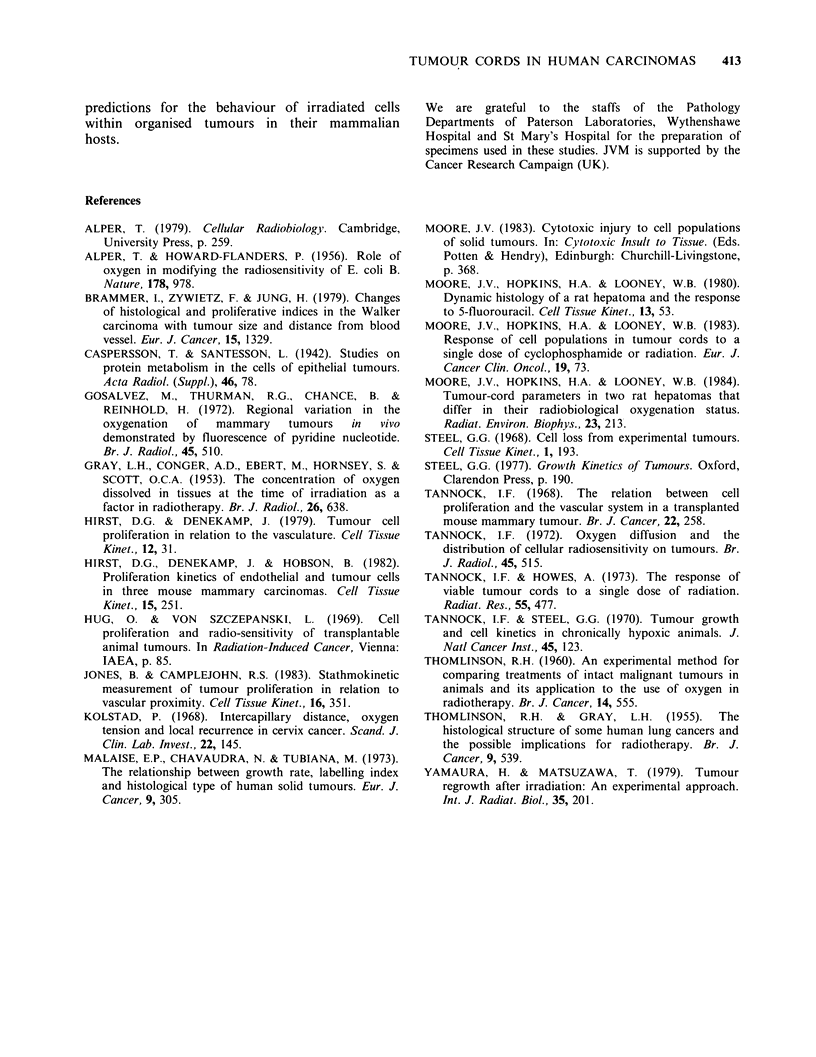# Tumour cords in 52 human bronchial and cervical squamous cell carcinomas: inferences for their cellular kinetics and radiobiology.

**DOI:** 10.1038/bjc.1985.55

**Published:** 1985-03

**Authors:** J. V. Moore, P. S. Hasleton, C. H. Buckley

## Abstract

Tumour cords have been measured in 33 cases of squamous cell carcinoma (SCC) of the bronchus and 19 cases of SCC of the uterine cervix. The overall mean cord radius for SCC in both sites was 104 microns, similar to the overall mean for various tumours in rodents. For tumour cells adjacent to blood vessels in cords of SCC, the mean Mitotic Index was 2.1% and from this value a rapid potential doubling time could be inferred (approximately 31 to 66 h). The proportion of dead cells within cords of cervical SCC was higher than in animal tumours. Using measured values for cord radius and published equations that describe the diffusion and consumption of oxygen in metabolising tissue, an attempt was made to calculate the oxygen partial pressure in vessels of cords of these SCC.


Br. J. Cancer (1985), 51, 407–413

### Tumour cords in 52 human bronchial and cervical squamous cell carcinomas: Inferences for their cellular kinetics and radiobiology

J.V. Moore[1], P.S. Hasleton[2] & C.H. Buckley[3]

[1]*Paterson Laboratories, Christie Hospital and Holt Radium Institute, Manchester M20 9BX;* [2]*Department of Pathology, Wythenshawe Hospital, Manchester M23 9LT;* [3]*Department of Pathology, St Mary's Hospital, Manchester M13 0JH, UK.*

**Summary** Tumour cords have been measured in 33 cases of squamous cell carcinoma (SCC) of the bronchus and 19 cases of SCC of the uterine cervix. The overall mean cord radius for SCC in both sites was 104 microns, similar to the overall mean for various tumours in rodents. For tumour cells adjacent to blood vessels in cords of SCC, the mean Mitotic Index was 2.1% and from this value a rapid potential doubling time could be inferred (~31 to 66 h). The proportion of dead cells within cords of cervical SCC was higher than in animal tumours. Using measured values for cord radius and published equations that describe the diffusion and consumption of oxygen in metabolising tissue, an attempt was made to calculate the oxygen partial pressure in vessels of cords of these SCC.

The parenchyma in the deeper-seated parts of a number of human and animal tumours grow in the form of multi-cell-layer, cylindrical cuffs that separate central blood vessels from areas of gross necrosis, or alternatively as spheroidal or rod-like structures with a central core of necrosis and a rim of healthy parenchyma, the whole surrounded by a basketwork of vessels. The term "tumour cord" has come to be associated primarily with the former type of feature but in the seminal paper of Caspersson & Santesson (1942) the term was applied to both types and this convention will be adopted here. Thomlinson & Gray (1955) analysed these structures in squamous cell carcinomas (SCC) of the human bronchus. They reported that the radial thicknesses of the healthy regions were relatively constant between tumours, and inferred, from considerations of oxygen concentration and metabolism, that the abrupt transition of cells from the histologically-intact to the necrotic compartment might occur by death of cells through lack of oxygen. This inference was of physiological interest but more importantly, of potential relevance to the practice of radiotherapy. Thomlinson & Gray (1955) reasoned that the apparently intact cells adjacent to gross necrosis would be severely hypoxic and therefore resistant to the action of ionising radiation (e.g. Gray *et al.*, 1953). Since these initial observations, a number of studies have been made on tumour cords in rodents, examining their cellular kinetics (Tannock, 1968; Hirst *et al.*, 1982), and the response to radiation (Tannock & Howes, 1973; Moore *et al.*, 1983) and to chemotherapy (Moore *et al.*, 1980, 1983). From the cellular kinetics of untreated experimental tumours, it may be inferred that a population of rapidly-dividing cells adjacent to the blood vessel (stem cells?), gives rise to daughters that are displaced away from the vessel, progressively lose their capacity to proliferate, and die. This "hierarchical" organisation presents the opportunity to analyse the histopathology of tumours in time and space, in a way more meaningful than random scoring of mitotic, "live" and "dead" cells. There is very little information in the literature by which one can judge the quantitative relevance of the experimental models to the corresponding structures in man. We present here data for tumour cords in squamous cell carcinomas of the human bronchus and uterine cervix.

Correspondence: J.V. Moore
Received 12 October 1984; and in revised form, 3 December 1984.

### Materials and methods

Gross samples of tumour were removed from fresh hysterectomy or pneumonectomy specimens, fixed in buffered, 10% formol-saline and processed routinely for histology. Five-$\mu$m-thick sections were cut, and stained with haematoxylin and eosin. Samples from the uterine cervix had been reported on (by CHB) as being SCC of the cervix, while samples from the lung had been classified (by PSH) as SCC of the bronchus. For SCC of the cervix, examination for the presence of tumour cords was made on slides of tumours from 140 successive patients, operated on between 1979 and 1983. For

© The Macmillan Press Ltd., 1985

SCC of the bronchus, the pathology reports routinely noted the presence of obvious focal or gross necrosis. Examination for cords was restricted to sections of tumours for which such mention had been made, a total of 96 patients operated on in 1982 and 1983.

The material examined ranged from poorly- to well-differentiated tumours. In some cases, it was difficult to distinguish between cords terminating in necrosis, and squamous differentiation that resulted in poor-quality parakeratin. An increase in spacing between cell nuclei, flattening of the nuclei and granulation in a prominent cytoplasm, were taken as evidence of residual differentiation. Such specimens were excluded from analysis, as also were patients who had received prior radiotherapy or chemotherapy. Measurements on tumour cords were made where a capillary was transected longitudinally and where the capillary lining and the row of pyknotic cells adjoining the gross necrosis, were approximately parallel. Parameters measured in each cord were:

(i) Cord "radius"; the radial distance between the capillary endothelium and the first pyknotic cell encountered at the intact/necrotic interface. Fifty cords were measured in each microscope slide.

For further analysis, the cord was then divided arbitrarily into zones of area 92 $\mu$m (parallel to the blood vessel) by 18 $\mu$m (at right angles to the vessel). Zone 1 adjoined the vessel, zones 2, 3 etc. were progressively nearer the necrosis.

(ii) Mitotic Index (MI); the number of cells in mitosis, divided by the total number of histologically-intact cells, in each zone.
(iii) Necrotic Index (NI); the number of histologically-dead cells (with pyknotic or karyorrhectic nuclei) divided by the total number of intact plus dead cells, in each zone.

For calculation of MI and NI, 50 cords were scored for each tumour.

### Results

From sections of cervical SCC in 140 patients, only 19 (14%) were adjudged to contain tumour cords. The mean age of these 19 patients was 42 years (range 27 to 68 years). For bronchial SCC, cords were found in tumours from 33 patients (34%). Twenty-eight (85%) were males (mean age 64 years, range 36 to 77), five were females (mean age 62, range 50 to 70). The mean number of tumour blocks taken from each operation specimen (both sites) was 3 (range 1 to 9).

*Cord radius*

For SCC cervix, mean values for cord thickness ranged from 42 to 152 $\mu$m (Figure 1). Where several samples had been taken from one tumour, average values of cord radius were obtained for each of these samples, and then overall means were calculated. One standard error on this grand mean was 5–10%, which suggests that for the tumours examined one tumour site was representative of the

**Figure 1** Radial thickness of cords in tumours of rodents and man. Numbers and open symbols are means ± s.e. for each tumour-line. For clarity, some of the central values have been omitted for SCC bronchus. Numbers accompanied by a query are for reports in which a precise value for radius was not quoted, but for which an approximation could be made. Such values were not included in the calculation of mean values ± 1 s.e. for all tumours in each of the three different species (shown as solid symbols). Numbers are for cords in: **mice** – 1. T50/80 mammary ca (Moore, 1983); 2. KHH mammary ca, (Hirst & Denekamp, 1979); 3. CA RH mammary ca, 4. KHU mammary ca (Hirst *et al.*, 1982); 5. BICR/SA1 mammary ca (Tannock, 1968); 6. Mammary ca (Tannock & Howes, 1973); 7. Mammary ca (Jones and Camplejohn, 1983); 8. Mammary ca (Gosalvez *et al.*, 1972). **Rats** – 9. 3924A hepatoma, 10. H-4-II-E hepatoma (Moore *et al.*, 1984); 11. AH109A hepatoma (Yamaura & Matsuzawa, 1979); 12. RIB5 fibrosarcoma (Thomlinson, 1960); 13. LMC1 mammary ca (Moore, unpublished); 14. Walker 256 carcinosarcoma (Brammer *et al.*, 1979); 15. Walker 256 (Hug & Szczepanski, 1969). **Man** – 16. Ca bronchus (Thomlinson & Gray, 1955).

whole. The mean cord radius for all the cervical SCC was $104 \pm 7\,\mu m$. For bronchial SCC, mean cord thickness ranged between 65 and 160 microns, the mean for all tumours being $104 \pm 5\,\mu m$ (Figure 1).

*Cell density*

There was 15% more cells per zone for bronchial SCC than cervical SCC, with relatively constant numbers across the different zones of the cords (Figure 2a).

*Mitotic index*

The relationship of MI to distance of a cell population from the subtending capillary, was that highest MI occurred near the vessel ($\approx 2.1\%$ for both bronchial and squamous SCC), least near the necrosis ($<0.5\%$; Figure 2b).

*Necrotic index*

For both sets of human tumours, the proportion of necrotic cells within the otherwise histologically-intact parenchyma of cords increased with increasing distance from the capillary. However, the values for cervical SCC were markedly higher than for bronchial SCC: by a factor of 4 in zone 1, 2 in zone 5 (Figure 2c).

**Discussion**

As noted by Hirst *et al.* (1982), the thickness of a tumour cord may be the resultant of the number of cells within the cord and their metabolic rate, and this might account for the systematic differences in cord thickness between tumours (Figures 1 and 2a). The high values for cord thickness in human bronchial SCC (mean $169\,\mu m$) reported by Thomlinson & Gray (1955) and which can be inferred for cervical SCC ($\sim 175\,\mu m$) from the colposcopy data of Kolstad (1968) relative to cords in rodents, have generally been ascribed to differences in metabolic rate of tumour cells in the different species. Our results for cord thickness in bronchial and cervical SCC yielded average values much lower than in the two previous reports for man, and only slightly higher than the average for 5 tumour lines in rats (by 7%) and 7 tumour lines in mice (by 12%). One obvious explanation for the differences between the two sets of results for SCC cervix, is that the present data are for formalin-fixed, wax-embedded material in which some shrinkage will have occurred, whilst 'the observations of Kolstad (1968) were made on tumours *in situ,* albeit by a method that does not have the same quantitative resolution as

**Figure 2** (a) Numbers of histologically-intact cells as a function of distance from the subtending vessel of cords in SCC bronchus (open circles, solid line) and cervix (open squares, solid line), and best fits to similar data for three experimental tumours (dashed lines, numbers keyed to the legend of **Figure 1**). (b) Mitotic Index as a function of distance from vessel. Symbols, etc., as for Figure 2(a). (c) Necrotic Index as a function of distance from vessel.

measurements of cords in thin sections. Both sets of results for bronchial SCC were for histologically-processed material. Here, the large differences in reported average cord thickness are accounted for partly by differences in data analysis. Thomlinson & Gray (1955) appear to have taken the widest cord in each of the 5 tumours they analysed and obtained an average of these ($169 \pm 8\,\mu m$). If instead, one uses the best-fit lines that they drew through their data, an average cord thickness of $134 \pm 7\,\mu m$ is obtained. The latter method was essentially the one used in the present report. The "widest cord" method may "compensate" somewhat for any (unknown) degree of shrinkage but ignores variability within an individual tumour (to be discussed later).

The untreated tumour cord in neoplasms of experimental animals has been shown to be a dynamic structure with a "flow" of cells away from, and predominantly at right angles to, the path of the subtending capillary (e.g. Tannock, 1968; Hirst *et al.*, 1982; Moore *et al.*, 1984). The tumour cell population next to the vessel is the ultimate source of this flow (although it is sustained by *all* mitoses) and is characterised by a high proportion of cells in cell cycle (mean growth fraction, i.e. the ratio of new proliferating cells to all new cells, for 5 rodent tumour lines was $79 \pm 13\%$; Table I) and a rapid rate of cell division (average of the mean cell cycle times for 4 rodent tumour lines was $17 \pm 1$ h; Table I). The potential doubling time (Tpot) of these populations which are assumed here to be in exponential growth, can be calculated by $Tpot = \ln 2 . Tm/MI$ or $Tpot = \lambda Ts/LI$ (from Steel, 1968), where Tm and Ts are the durations of the mitotic and DNA-synthetic phases of the cell cycle respectively, MI and LI the mitotic and pulse-[$^3$H] thymidine labelling indices respectively, and $\lambda$ a constant for a given tumour.

For 8 rodent tumour-lines, mean Tpot for these inner zones of cords was $25.9 \pm 5$ h (Table I). This calculation has been made for zone 1 of cords in bronchial and cervical SCC, assuming Tm to be either 1 or 2 h. Taking the mean MI in each case, Tpot was 33 or 66 h for cervical SCC and 31 or 63 h for bronchial SCC (Table I). Although longer than for the rodent tumours, these values of Tpot still imply rapid proliferation among the cells that, in a restricted sense, can be regarded as the stem cells of the cords in these human tumours. Malaise *et al.* (1973) collated data for mean cell cycle times (Tc) of 11 human SCC, from which an overall mean of $55 \pm 16$ h can be calculated. Using this value, an estimate of the Growth Fraction (GF) of cells in zone 1 of cords can be made, by:

$$\ln(1 + GF) = \ln 2. (Tc/Tpot) \text{ from Steel (1968).}$$

The lower calculated estimates of Tpot for cells of zone 1 in cords of SCC cervix and bronchus (33 and 31 h, respectively) yielded values of GF greater than 1, while the higher estimates (66 and 63 h) predicted GF of 78% and 83% respectively. The assumptions and approximations of these calculations are very considerable but it seems not unreasonable to conclude that the majority of cells adjacent to the capillaries in these human tumours are in rapid cycle, as in the rodent tumours.

A striking difference between rodent and human tumours lies in the volume doubling time (VDT) of the whole tumour. In the 8 rodent lines cited in Table I, VDT averaged $5.1 \pm 1.3$ days, for tumour sizes where cords were present. In contrast, Malaise *et al.* (1973) calculated an average doubling time of 58 days from several series of human SCC, including bronchus and cervix. This difference in the observed rate of growth is usually attributed to high rates of "cell loss" in human tumours (Steel, 1977). From the present data no meaningful quantitative comment can be made on cell loss, but it may be noteworthy that the proportion of dead cells within tumour cords of cervical SCC and to a lesser extent bronchial SCC, tended to be larger than those for the few rodent tumours in which such measurements have been made (Figure 2c).

As regards the radiobiology of tumour cords, Thomlinson & Gray (1955) and Tannock (1972) calculated the "critical radius" at which oxygen ($O_2$) tension should fall to zero, based on several assumptions as to partial pressure of $O_2$ in the blood vessels (taken to be 40 mm Hg), diffusion of $O_2$ through tissue, $O_2$ consumption of cells, etc. These generally-accepted calculations resulted in values for critical radii that accorded reasonably well with measured cord radii and led to the important inference that histologically-intact cells adjacent to the necrosis might be hypoxic to an extent that rendered them resistant to the action of ionising radiation. It is of interest to reverse these calculations, i.e. to use measured cord radii to calculate the "partial pressure of $O_2$" ("$pO_2$") in the subtending vessels and thence to calculate "$pO_2$" in the different zones of the cords in SCC cervix and bronchus. These estimations have been made for two geometric conditions: (i) diffusion of $O_2$ inward from vessels surrounding a spheroidal or rod-like cord (the predominant form observed in this series); or (ii) diffusion of $O_2$ outward from a central blood vessel. The diffusion equations employed were as given by Tannock (1972); the constants used were taken from Thomlinson & Gray (1955), including the major assumption that the cells of these tumours all had an $O_2$ consumption of $5.2 \mu l \, mg^{-1}$ dry weight $h^{-1}$.

A family of curves of "$pO_2$" versus distance from blood vessel, was plotted for the 19 cases of SCC cervix, for which the range of cord radii was slightly wider than for SCC bronchus. The two geometric models lead to very different predictions of "$pO_2$" in the blood vessels (Figure 3): for diffusion inward, the mean value for the 19 tumours was $22 \pm 3$ mm Hg (Figure 3a); for diffusion outward, the mean was $107 \pm 15$ mm Hg (Figure 3b). The latter value is higher than the $pO_2$ for non-tumour arterial blood ($\approx 95$ mm Hg) and seems particularly implausible for capillaries in the deeper-seated

**Table I** Parameters of cellular kinetics of corded tumours in rodents and man

| Tumour | *Cells adjacent to blood vessels in cords* | | | | Cord radius (μm) | Cord transit time (h) | Tumour volume doubling time (days) | Reference |
|---|---|---|---|---|---|---|---|---|
| | *MI%* | *Tc(h)* | *Tpot(h)* | *GF% (means)* | | | | |
| *Mouse* (mammary carcinomas) | | | | | | | | |
| BICR/SA1 | 3.1 | 16 | 26 | 100 | 85 | 36 | 3.2[a] | Tannock (1968) |
| KHH | 3.7 | 18 | 14 | 100 | 80 | 36 | 5.0 | Hirst & Denekamp (1979) |
| CA RH | — | — | 58[b] | | 97 | 50 | 13.3 | Hirst *et al.* (1982) |
| KHU | — | 16 | 22[b] | — | 57 | 50 | 6.0 | Hirst *et al.* (1982) |
| T50/80 | 2.3 | — | 22 | — | 99 | — | 6.4 | Moore (1983) |
| "C3H" | 2.4 | — | 24 | 36 | 80 | — | 1.7 | Jones & Camplejohn (1983) |
| *Rat* (hepatomas) | | | | | | | | |
| 3924A | 2.2 | — | 16 | 100 | 118 | 52 | 2.6 | Moore *et al.* (1984) |
| H-4-II-E | 2.8 | 17 | 25 | 60 | 69 | 40 | 2.6 | Moore *et al.* (1984) |
| *Man* (SCC) | Assumes (i) Tm = 1.0; (ii) Tm = 2 h. | | | | | | | |
| Cervix | 2.1 (range 0.9, 4.0) | — | (i) 33 (77, 17) (ii) 66 (154, 35) | | 104 | | | Present paper |
| Bronchus | 2.2 (range 1.2, 3.9) | | (i) 31 (57, 18) (ii) 63 (116, 36) | | 104 | | | Present paper |

[a]Mass doubling time.

[b]Value for whole tumour.

**Figure 3** (a) Calculated oxygen partial pressures in tumour tissue of cords in 19 different cervical SCC (descending lines) as a function of distance from peripheral blood vessels. Ascending line shows the expected relative radiosensitivity (upper abscissa) of tumour cells for which $m = 2.75$ and $k = 7.5$ mm Hg, as a function of oxygen partial pressure (ordinate). Dashed line links a "$pO_2$" of 7.5 mm Hg with a relative sensitivity of $[m+1]/2 = 1.9$. (b) As for Figure 3a, but assuming a cord geometry in which a cylinder of parenchyma surrounds a single central capillary.

regions of large tumours. As noted earlier, the first geometric model more closely accords with what was seen in the tumour sections. Adopting this model, only 3 of 19 tumours had "$pO_2$" values that correspond to the $pO_2$ for non-tumour venous blood ($\approx$40 mm Hg), the remainder ranging from 32 down to 3 mm Hg. Whether these rather low values are of potential relevance to radiotherapy, depends on the $pO_2$ that corresponds to significant radiobiological hypoxia, i.e. where the oxygen level is sufficiently low as to reduce the sensitivity of mammalian cells to the lethal effects of radiation. The relationship between radiosensitivity (S) and $pO_2$ for several neoplastic and non-neoplastic cell systems has been shown to conform to the equation described by Alper & Howard-Flanders (1956):

$$S/Sn = (m[pO_2] + k)/([pO_2] + k)$$

where Sn is the sensitivity in the absence of oxygen; m is the ratio of the maximum obtainable sensitivity in the presence of $O_2$, to Sn; and k is the partial pressure or concentration of $O_2$ that corresponds to a sensitivity half-way between the minimum and maximum (for populations whose response can be fitted by the above equation). Values of m range typically between 2.5 and 3, but estimates of k (made *in vitro*) vary rather widely between about 2 and 12 mm Hg (Alper, 1979). For purposes of illustration, we have arbitrarily adopted values of 2.75 for m and 7.5 mm Hg for k. First-order estimates can then be made of the possible radiobiological status of cell populations in spheroidal or rod-like cords of the 19 SCC of cervix (Figure 3a) In tumours of 2/19 patients, the rapidly-dividing cells lying on average 9 μm from the surrounding vessels, would have a relative radiosensitivity (r) half-maximal or less (i.e. $< 1.9$, where $r = [m+1]/2$). For cells an average of 27 μm away, this reduced radiosensitivity would occur in 32% of the tumours (6/19); at 45 μm, in 58%; 63 μm, 74%; 81 μm, 89%, 99 μm, 100%. Thus under the conditions specified, all these tumours would be expected to contain histologically-intact cells that should be relatively resistant to the lethal action of ionising radiation. In 2 cases this would occur among cell populations which one might reasonably expect to be particularly rich in clonogenic cells – near the vessels. It should be noted also that these calculations have been made using *mean* values of cord radius, from a sample of 50 cords for each tumour. For all tumours with a mean cord radius of 100 μm, the lower boundary of the *range* was ~45 μm. This admits of the possibility that within a tumour whose zone 1 cells were calculated to be well-oxygenated on average, there existed one or more cords whose rapidly-dividing "stem cells" might be radiobiologically less than fully oxygenated.

The numerous assumptions of these calculations are readily acknowledged. However, it has been shown for one tumour-line that cord radius is sensitive to alterations in the $O_2$ milieu (by lowering the $O_2$ tension in the air respired by the host; Tannock & Steel, 1970). while values of cord radius, MI and NI in 2 rat hepatomas accord qualitatively with their relative oxygenation status, radiobiologically – defined (Moore *et al.*, 1984). The cord model as proposed by Thomlinson and Gray (1955) represents a unique synthesis of tumour pathology, physiology and radiobiology, and it is perhaps surprising that more attempts have not been made over the years to test its predictions for the behaviour of irradiated cells within organised tumours in their mammalian hosts.

We are grateful to the staffs of the Pathology Departments of Paterson Laboratories, Wythenshawe Hospital and St Mary's Hospital for the preparation of specimens used in these studies. JVM is supported by the Cancer Research Campaign (UK).

### References

ALPER, T. (1979). *Cellular Radiobiology*. Cambridge, University Press, p. 259.

ALPER, T. & HOWARD-FLANDERS, P. (1956). Role of oxygen in modifying the radiosensitivity of E. coli B. *Nature*, **178**, 978.

BRAMMER, I., ZYWIETZ, F. & JUNG, H. (1979). Changes of histological and proliferative indices in the Walker carcinoma with tumour size and distance from blood vessel. *Eur. J. Cancer*, **15**, 1329.

CASPERSSON, T. & SANTESSON, L. (1942). Studies on protein metabolism in the cells of epithelial tumours. *Acta Radiol.* (*Suppl.*), **46**, 78.

GOSALVEZ, M., THURMAN, R.G., CHANCE, B. & REINHOLD, H. (1972). Regional variation in the oxygenation of mammary tumours *in vivo* demonstrated by fluorescence of pyridine nucleotide. *Br. J. Radiol.*, **45**, 510.

GRAY, L.H., CONGER, A.D., EBERT, M., HORNSEY, S. & SCOTT, O.C.A. (1953). The concentration of oxygen dissolved in tissues at the time of irradiation as a factor in radiotherapy. *Br. J. Radiol.*, **26**, 638.

HIRST, D.G. & DENEKAMP, J. (1979). Tumour cell proliferation in relation to the vasculature. *Cell Tissue Kinet.*, **12**, 31.

HIRST, D.G., DENEKAMP, J. & HOBSON, B. (1982). Proliferation kinetics of endothelial and tumour cells in three mouse mammary carcinomas. *Cell Tissue Kinet.*, **15**, 251.

HUG, O. & VON SZCZEPANSKI, L. (1969). Cell proliferation and radio-sensitivity of transplantable animal tumours. In *Radiation-Induced Cancer*, Vienna: IAEA, p. 85.

JONES, B. & CAMPLEJOHN, R.S. (1983). Stathmokinetic measurement of tumour proliferation in relation to vascular proximity. *Cell Tissue Kinet.*, **16**, 351.

KOLSTAD, P. (1968). Intercapillary distance, oxygen tension and local recurrence in cervix cancer. *Scand. J. Clin. Lab. Invest.*, **22**, 145.

MALAISE, E.P., CHAVAUDRA, N. & TUBIANA, M. (1973). The relationship between growth rate, labelling index and histological type of human solid tumours. *Eur. J. Cancer*, **9**, 305.

MOORE, J.V. (1983). Cytotoxic injury to cell populations of solid tumours. In: *Cytotoxic Insult to Tissue*. (Eds. Potten & Hendry), Edinburgh: Churchill-Livingstone, p. 368.

MOORE, J.V., HOPKINS, H.A. & LOONEY, W.B. (1980). Dynamic histology of a rat hepatoma and the response to 5-fluorouracil. *Cell Tissue Kinet.*, **13**, 53.

MOORE, J.V., HOPKINS, H.A. & LOONEY, W.B. (1983). Response of cell populations in tumour cords to a single dose of cyclophosphamide or radiation. *Eur. J. Cancer Clin. Oncol.*, **19**, 73.

MOORE, J.V., HOPKINS, H.A. & LOONEY, W.B. (1984). Tumour-cord parameters in two rat hepatomas that differ in their radiobiological oxygenation status. *Radiat. Environ. Biophys.*, **23**, 213.

STEEL, G.G. (1968). Cell loss from experimental tumours. *Cell Tissue Kinet.*, **1**, 193.

STEEL, G.G. (1977). *Growth Kinetics of Tumours*. Oxford, Clarendon Press, p. 190.

TANNOCK, I.F. (1968). The relation between cell proliferation and the vascular system in a transplanted mouse mammary tumour. *Br. J. Cancer*, **22**, 258.

TANNOCK, I.F. (1972). Oxygen diffusion and the distribution of cellular radiosensitivity on tumours. *Br. J. Radiol.*, **45**, 515.

TANNOCK, I.F. & HOWES, A. (1973). The response of viable tumour cords to a single dose of radiation. *Radiat. Res.*, **55**, 477.

TANNOCK, I.F. & STEEL, G.G. (1970). Tumour growth and cell kinetics in chronically hypoxic animals. *J. Natl Cancer Inst.*, **45**, 123.

THOMLINSON, R.H. (1960). An experimental method for comparing treatments of intact malignant tumours in animals and its application to the use of oxygen in radiotherapy. *Br. J. Cancer*, **14**, 555.

THOMLINSON, R.H. & GRAY, L.H. (1955). The histological structure of some human lung cancers and the possible implications for radiotherapy. *Br. J. Cancer*, **9**, 539.

YAMAURA, H. & MATSUZAWA, T. (1979). Tumour regrowth after irradiation: An experimental approach. *Int. J. Radiat. Biol.*, **35**, 201.